# Supplementary material for: Recent Clinical Isolates of Enterovirus D68 Have Increased Replication and Induce Enhanced Epithelial Immune Response Compared to the Prototype Fermon Strain
Source: Viruses. 2023 May 31;15(6):1291. doi: 10.3390/v15061291 (PMC10305710; doi:10.3390/v15061291)
Supplement: Supplementary file 1 [file viruses-15-01291-s001.zip › viruses-2374192-supplementary.pdf]

Supplementary materials

Supplementary methods

RNA-sequencing library construction and sequencing of directional libraries

For each library preparation, mRNA was purified from 200 ng of total RNA using poly-T oligo-attached magnetic beads. Subsequently, each poly-A enriched sample was fragmented using divalent cations under elevated temperature. The RNA was synthesized into double-stranded cDNA using SuperScript II Reverse Transcriptase (Invitrogen, Carlsbad, California, USA) and random primers for first strand cDNA synthesis followed by second strand synthesis. Double-stranded cDNA was purified by paramagnetic beads (Agencourt AMPure XP beads, Beckman Coulter). The cDNA products were incubated with Klenow DNA Polymerase to add an ‘A’ base (Adenine) to the 3’ end of the blunt DNA fragments. DNA fragments were ligated to Illumina adapters. The adapter-ligated DNA products were purified by paramagnetic beads. Adapter ligated DNA was amplified in a Linker Mediated PCR reaction (LM-PCR) for 11 cycles using Phusion™ DNA Polymerase and Illumina’s PE genomic DNA primer set and then purified by paramagnetic beads. Quality and quantity of the finished libraries were assessed using an Agilent HS DNA or DNA1000 chip (Agilent Technologies, Inc., Santa Clara, CA, USA) and Qubit® dsDNA HS Assay Kit (Invitrogen, Carlsbad, California, USA), respectively. Libraries were standardized to 2 nM. Cluster generation was performed using standard Cluster Kits (v4) and the Illumina cBot. Single end 100 bp sequencing was performed, using standard SBS chemistry (v4) on an Illumina HiSeq2500 sequencer. Images were analyzed using the standard Illumina Pipeline, version 1.8.2.

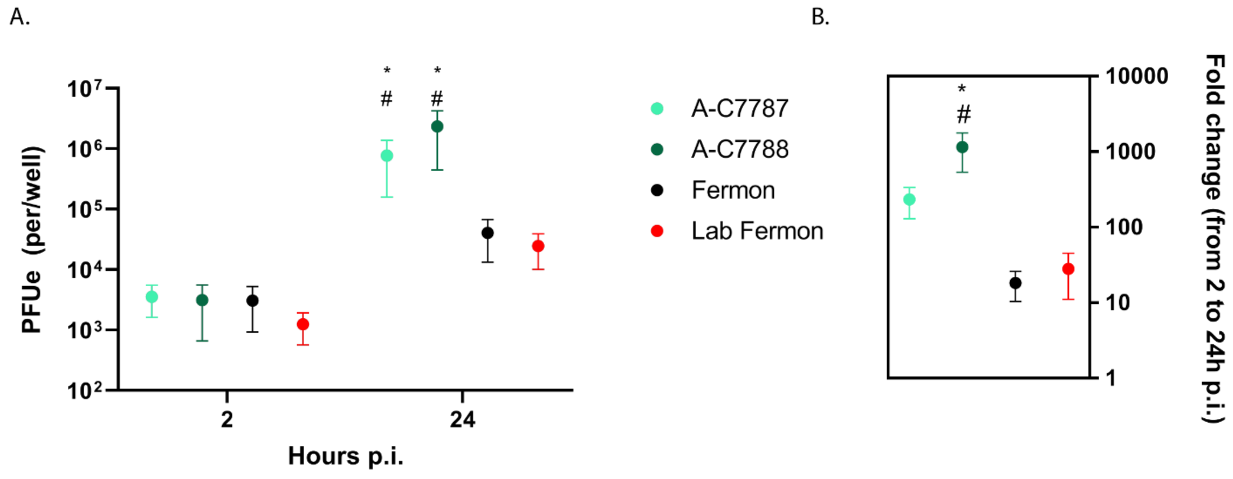

Supplementary Figure S1. Comparison of viral binding and replication of two Fermon strains and two recent EV-D68 clinical isolates in PBE-ALI cells. Fermon strain provided by Dr. Frank van Kuppeveld (Utrecht University) is referred to as "Fermon" and Fermon strain that was cloned in our laboratory using published sequence (GenBank accession # AY426531) is referred to as "Lab Fermon". (**A**) Viral binding (2h p.i.) and progeny yields (24h p.i.) and (**B**) viral RNA replication of 2 Fermon variants and 2 recent clinical isolates that belong to B1 phylogenetic clade. * : $p<0.05$ vs Lab Fermon. #: $p<0.05$ vs Fermon.

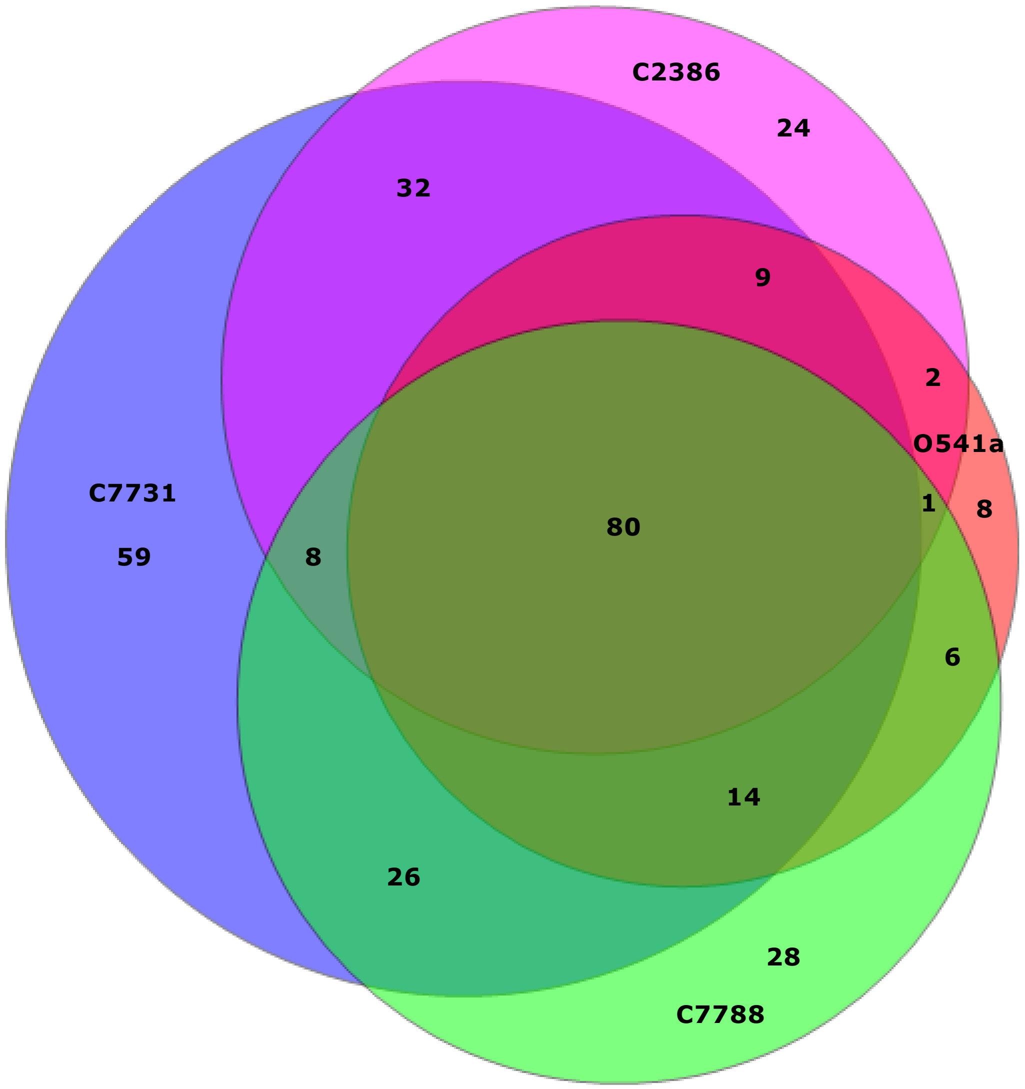

Supplementary Figure S2. Area-proportional Venn diagram depicting genes induced by recent EV-D68 isolates in PBE-ALI cells. The differentially expressed (DE) genes (FDR <0.2; 1.5-fold upregulated) induced by each isolate were compared. The overlapping segments of the circles with numbers represent common genes. The diagram was created using an online tool DeepVenn (https://www.deepvenn.com/) [Hulsen, 2022][1].

Reference

1. Hulsen, T.; de Vlieg, J.; Alkema, W. Biovenn - a web application for the comparison and visualization of biological lists using area-proportional venn diagrams. *BMC Genomics* **2008**, *9*, 488.
